# From Light Microscopy to Analytical Scanning Electron Microscopy (SEM) and Focused Ion Beam (FIB)/SEM in Biology: Fixed Coordinates, Flat Embedding, Absolute References

**DOI:** 10.1017/S1431927618015015

**Published:** 2018-09-24

**Authors:** Manja Luckner, Gerhard Wanner

**Affiliations:** Department Biology I, Ultrastructural Research, Ludwig-Maximilians-University Munich, 82152 Planegg-Martinsried, Germany

**Keywords:** CLEM, EDX, FIB-SEM, flat embedding, tomography

## Abstract

Correlative light and electron microscopy (CLEM) has been in use for several years, however it has remained a costly method with difficult sample preparation. Here, we report a series of technical improvements developed for precise and cost-effective correlative light and scanning electron microscopy (SEM) and focused ion beam (FIB)/SEM microscopy of single cells, as well as large tissue sections. Customized coordinate systems for both slides and coverslips were established for thin and ultra-thin embedding of a wide range of biological specimens. Immobilization of biological samples was examined with a variety of adhesives. For histological sections, a filter system for flat embedding was developed. We validated ultra-thin embedding on laser marked slides for efficient, high-resolution CLEM. Target cells can be re-located within minutes in SEM without protracted searching and correlative investigations were reduced to a minimum of preparation steps, while still reaching highest resolution. The FIB/SEM milling procedure is facilitated and significantly accelerated as: (i) milling a ramp becomes needless, (ii) significant re-deposition of milled material does not occur; and (iii) charging effects are markedly reduced. By optimizing all technical parameters FIB/SEM stacks with 2 nm iso-voxels were achieved over thousands of sections, in a wide range of biological samples.

## Introduction

**Figure 1:**
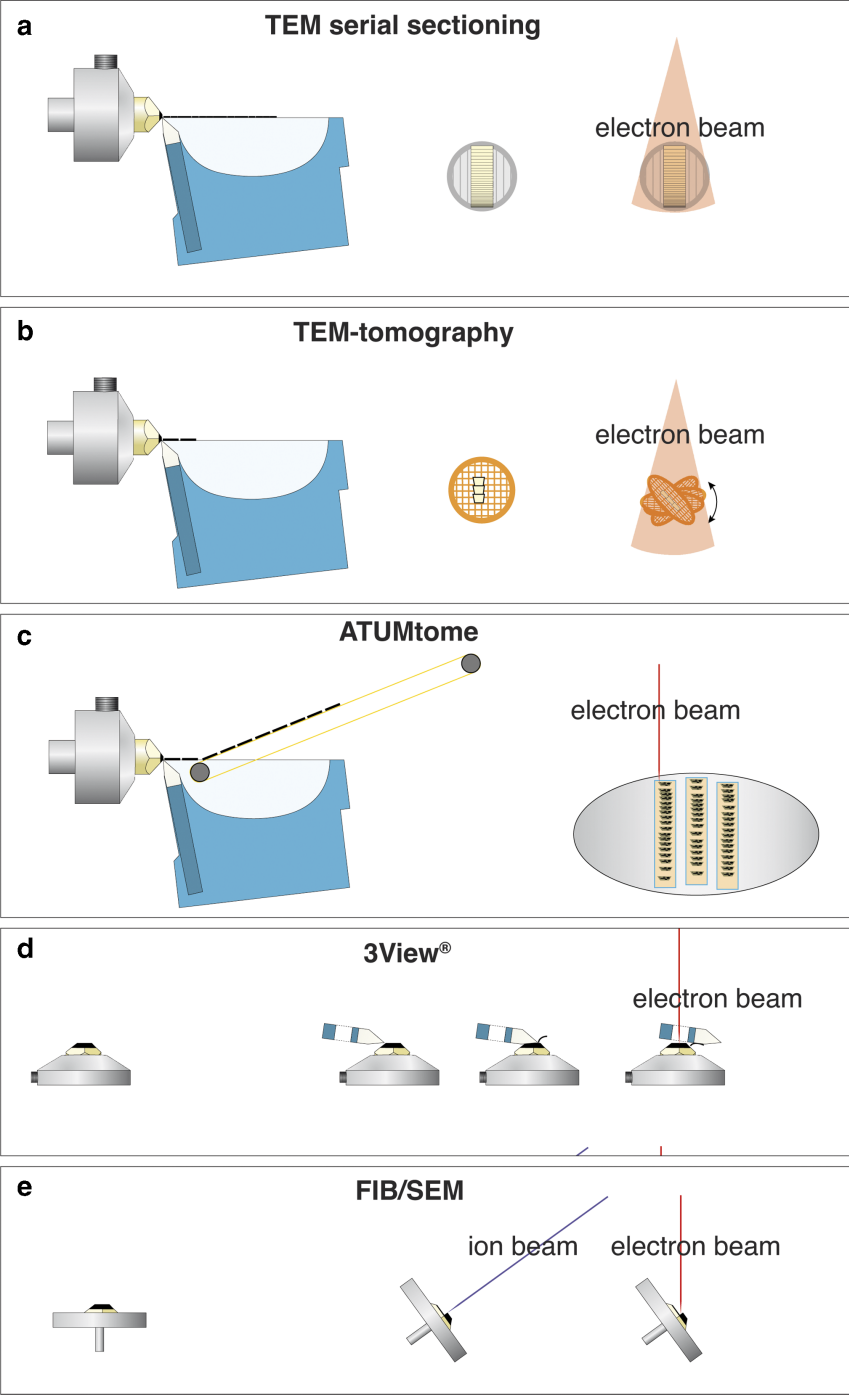
Comparison of volume electron microscopy techniques, for classical transmission EM (TEM) serial sectioning, consecutive ultrathin sections are collected on grids and imaged separately with TEM (**a**). For TEM-tomography thin sections (0.3–1 *µ*m) are cut with a diamond knife, collected onto a grid, which is tilted relative to the TEM to 70°. After registration and back-projection, a tomogram is provided (**b**). For array-tomography serial sections are cut with a diamond knife, collected with an automated conveyor belt onto an adhesive tape (ATUMtome), mounted onto glass slides or silicon wafers and investigated with an scanning electron microscopy (SEM) (**c**). 3View®, the commercial version of the invention of W. Denk, manufactured by Gatan with a built-in ultramicrotome within the SEM. Serial sections are cut with a moving diamond knife and the block-face is imaged after every section (**d**). Focused ion beam (FIB)/SEM serial block-face milling is achieved by tilting a specimen in an SEM to 54°. An ion-gun is placed in the SEM at the same angle so that sections can be milled orthogonal to the specimen surface. Block-face images are taken at an angle of 36° with an SEM, and either backscattered electrons or secondary electrons are detected (**e**).

For three-dimensional (3D) ultrastructural investigations with electron microscopy (EM), five different techniques are typically used (Fig. 1). Each one of these techniques, however, has significant drawbacks: (i) classical transmission EM (TEM) serial sectioning, which is largely unsuitable for long series, has many drawbacks, such as compression of sections, uneven stretching, folds, knife marks, etc.; (ii) TEM-tomography, although achieving the highest resolution, is limited in section thickness (max. 1 *µ*m); (iii) serial block face sectioning (3View^®^), a built-in ultramicrotome within a scanning electron microscopy (SEM), is suitable for large volumes but limited in section thickness (20 nm at best) and is hampered by charging; (iv) array tomography, which is non-destructive, but limited in *z*-resolution just as classical serial sectioning; and (v) focused ion beam (FIB)/SEM-tomography, which currently offers by far the highest resolution along the *z* axis with a “section thickness” down to 2 nm for long image series (for review see: Peddie & Collinson, 2014; Romero-Brey & Bartenschlager, 2015; Karreman et al., 2016; Xu et al., 2017). A period of 10 years after FIB/SEM was recognized as a revolutionary tool for 3D-EM in biology, these instruments are widely accepted as expensive, but highly complementary and efficient tools, which are indispensable for ultrastructural studies. However, the analytical potential of the instrument itself, the SEM, is still not fully exploited. Initially, a SEM was simply considered a surface imaging instrument. However, the use of different detectors and the variation of many SEM parameters offer enormous analytical capacities, well beyond surface imaging.

Three different signals can be routinely used for structural and analytical information, as described by Bozzola & Russell (1991). First, the secondary electrons (SE) give the characteristic topography of SEM images. Second, the backscattered electrons (BSE) provide a material contrast and due to their high energy and sub-surface information. Third, X-ray detection can be harnessed to analyze the local atomic composition (Scala et al., 1985; Bozzola & Russell, 1991; Utke et al., 2012; Drobne, 2013). These signals can be monitored separately or in combination and are indispensable for SEM and to some extent required for FIB/SEM.

Despite utilizing multiple signals, a correlation of light microscopy (LM) and EM remains challenging. Reference points or fiducial markers are essential to facilitate a correlation, but a universal labeling does not exist, since the requirements vary widely for different experiments. Many years before they were commercially available, we developed slides (=point finder) with stable, solvent resistant coordinates by sintering transfer pictures (decalcomania; Wanner et al., 1993). Since the labeling was elevated, experiments such as chromosome spreads were impeded. With new laser technology, it was possible to produce engraved coordinate systems with much finer symbols. These slides were suitable for wider range of biological samples, including chromosome spreads, microorganisms, and proliferating cultured cells. Confocal laser scanning microscopy has broadened the spectrum allowing the production of labeled coverslips.

**Figure 2:**
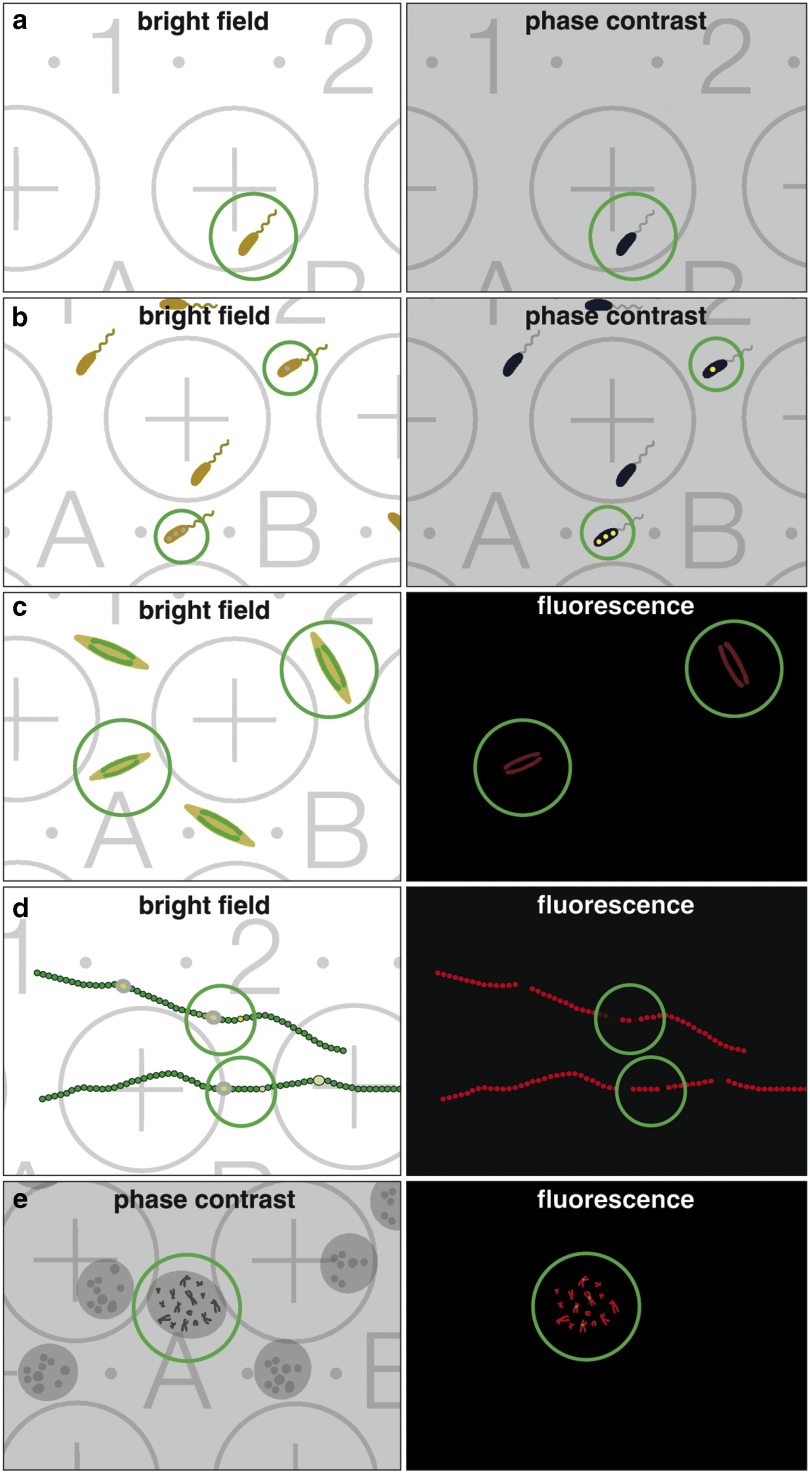
Benefit of a coordinate system for correlative light and electron microscopy. Examples of common investigations, which are either facilitated or made possible by correlative light microscopy (LM)/scanning electron microscopy (SEM). The classical claim, identifying rare objects in the LM with bright field or phase contrast (**a**; circle) and simply re-locating in SEM. Cells often produce inclusion bodies, not seen in the bright field but in phase contrast (**b**; circles), can be further analyzed in SEM. Vitality is an important criterion; cells (e.g. diatoms) with chloroplasts show autofluorescence in living state (**c**; circles). Non-fluorescent cells can be ignored for further investigation with SEM. For any image interpretation, oriented section planes of biological structures are essential; e.g. heterocysts of filamentous cyanobacteria can be investigated with LM, selected based on vitality of the vegetative cells (autofluorescence of chlorophyll) and presence of heterocysts, documented with the coordinates and re-located in SEM for either cross or longitudinal sections with focused ion beam (**d**; circles). Rare cytological targets, as specific labeled chromosomes, are investigated/selected with phase contrast and fluorescent signals to enable further SEM investigations (**e**; circle).

When correlative light and electron microscopy (CLEM) became fashionable, the scientific interest was primarily focused on improving LM resolution, aiming to image the smallest structures first in live mode, and afterwards complementing these images with ultrastructural information. Unfortunately, the term CLEM is usually associated with recent studies using state of the art high-resolution 3D-LM. However, many studies only require a rather simple correlation with low-resolution wide-field/fluorescence LM, which is illustrated for some examples (Fig. 2), highlighting the importance of coordinates facilitating CLEM, e.g. (i) finding rare cells growing in low density (Fig. 2a); (ii) finding cells with inclusions (Fig. 2b); (iii) selecting vital cells of a population (Fig. 2c); (iv) registering heterocysts of filamentous cyanobacteria, either for cross or longitudinal FIB/SEM milling (Fig. 2d); or (v) selecting immunolabeled chromosomes in metaphase (Fig. 2e).

A critical requirement for CLEM is firmly maintaining the sample in position, thus maintaining the orientation from live imaging LM to SEM. As only a minor fraction of biological specimens are inherently adherent or sticky (e.g. adherent cell cultures), it is necessary to establish procedures to immobilize a wide range of biological objects, such as single cells, cell aggregates, tissues, or tissue sections. Sample adhesion has to be stable to withstand all changes of solutions, buffers, fixatives, and solvents both by plunging or centrifugation. Finally, a prerequisite for correlative microscopy consists of embedding specimens in very thin layers of epoxy resin for precise re-localization of regions of interest (ROIs). Depending on cell size, density, etc., it is desirable to control the thickness of the sample with regards to the requirements of the scientific question. The first attempts for flat embedding were achieved by draining the resin by gravity, centrifugation, or careful blotting (Kizilyaprak et al., 2014). In another study, animals infiltrated in 100% resin were removed from the resin droplet using toothpicks or pins, moved onto the substrate and drained with filter paper (Schieber et al., 2017). To significantly reduce the final resin layer, cells were infiltrated with 100% resin, then quickly rinsed with 100% ethanol to remove of excess resin (Belu et al., 2016). In a recent book chapter, the technical possibilities for various embedding protocols (classical *en bloc* embedding and thin-layer plastification) were compared for live cell imaging of adherent cells with volume SEM using Ibidi *µ*-dish 500 or MatTek finder grid dishes. Minimal resin covering of cells was achieved by upright positioning of the cell substrate for draining and with polymerization starting at lower temperatures (Lucas et al., 2017).

Our aim was to enable CLEM, by avoiding any hazardous or complicated manipulations, to evolve from an expensive method into a cost-effective technology suitable for a wide spectrum of biological samples. To this end, we focused on the following aims: (i) to design and produce slides and coverslips with a variety of customized coordinates for correlative LM and SEM and FIB/SEM of both critical point dried (CPD) and flat embedded samples; (ii) to establish a labeling technique for “post-embedding”; (iii) to evaluate strategies to immobilize cells and tissue sections; (iv) to develop a filter system for “flat embedding” of large, fragile or delicate specimens; and (v) to use thick epoxy sections for high-resolution LM, TEM, and FIB/SEM tomography. Each topic was validated for at least one scientific challenge.

## Material and Methods

### Manufacturing of Customized Coordinates

#### Elevated Labels for Slides and Cover Slips

Coordinates, which are elevated and added on top of the surface of the slides/cover slips, were made by Gaßner Glastechnik GmbH (Planegg, Germany) with transfer pictures (decalcomania), glued to the slide and sintered at high temperature (Point finder; Wanner et al., 1990, 1993). AG Lasergravuren (Weilheim, Germany) produced extremely dense and small labels with sintered titanium on cover slips.

#### Engraved Slides and Cover Slips

Laser engravings on slides or coverslips were produced by Laser Marking, (Fischen, Germany), Grüner Laser Products GmbH & Co. KG (Munich, Germany) and AG Lasergravuren (Weilheim, Germany), according to our desired coordinate systems template.

#### Water-Resistant Stamp

A water-resistant stamp with a coordinate system according to our design was manufactured by modico GmbH & Co KG (Fürstenfeldbruck, Germany).

### Biological Material

Chromosomes were isolated, fixed with formaldehyde, and processed as described by Wanner & Schroeder-Reiter (2008) and Wanner et al. (2015). *M. bavaricum* (kindly provided by Prof. Dr. Dirk Schüler; University of Bayreuth) were fixed onto slides either by drop-cryo preparation (Wanner et al., 2008) or by high-pressure freezing as described by Jogler et al. (2011). *Anabaena catenula* (strain SAG 1403-1; EPSAG, Göttingen, Germany) was fixed with 2.5% glutaraldehyde in cacodylate buffer (2 mM NaCl; 2 mM MgCl_2_; 75 mM cacodylate; pH 7,0) or high-pressure frozen and freeze substituted before immobilization onto slides. *Porphyridium purpureum* was purchased from EPSAG, fixed with 2.5% glutaraldehyde in cacodylate buffer (2 mM NaCl; 2 mM MgCl_2_; 75 mM cacodylate; pH 7,0) and embedded into epoxy resin. *Tradescantia zebrina* was provided by the Botanical Garden (Munich, Germany) and fixed with 2.5% glutaraldehyde, 2 mM NaCl; 2 mM MgCl_2_; 75 mM cacodylate; pH 7,0. HeLa Kyoto cells were kindly provided by Prof. Dr. Heinrich Leonhardt (LMU, Munich, Germany). Cells were cultured and grown on laser marked slides and fixed as described by Luckner & Wanner (2018). Human platelets were cultured and immunolabeled by Dr. Florian Gärtner (Klinikum, LMU) (Gaertner et al., 2017). Breast cancer cells (SKBR3) were kindly provided by Prof. Dr. Angelika Vollmar (LMU) and fixed the same way as HeLa cells. Mouse brain tissue was kindly provided by Prof. Dr. Jochen Herms (DZNE, Munich, Germany) fixed with 2.5% glutaraldehyde in cacodylate buffer and post-fixed as described below.

### Coating of Slides with Adhesives

#### Poly-lysine (Merck, Darmstadt, Germany)

Laser marked slides/cover slips were coated with poly-lysine according to the manufacturer’s instructions. Poly-lysine coated slides were used for drop/cryo-fixation of chromosomes (Martin et al., 1994; Wanner & Schroeder-Reiter, 2008; Wanner et al., 2015), fixation of *M. bavaricum* (Jogler et al., 2011) and *Chlorochromatium aggregatum* (Wanner et al., 2008).

#### Biobond (Science Service GmbH, Munich, Germany)

**Figure 3:**
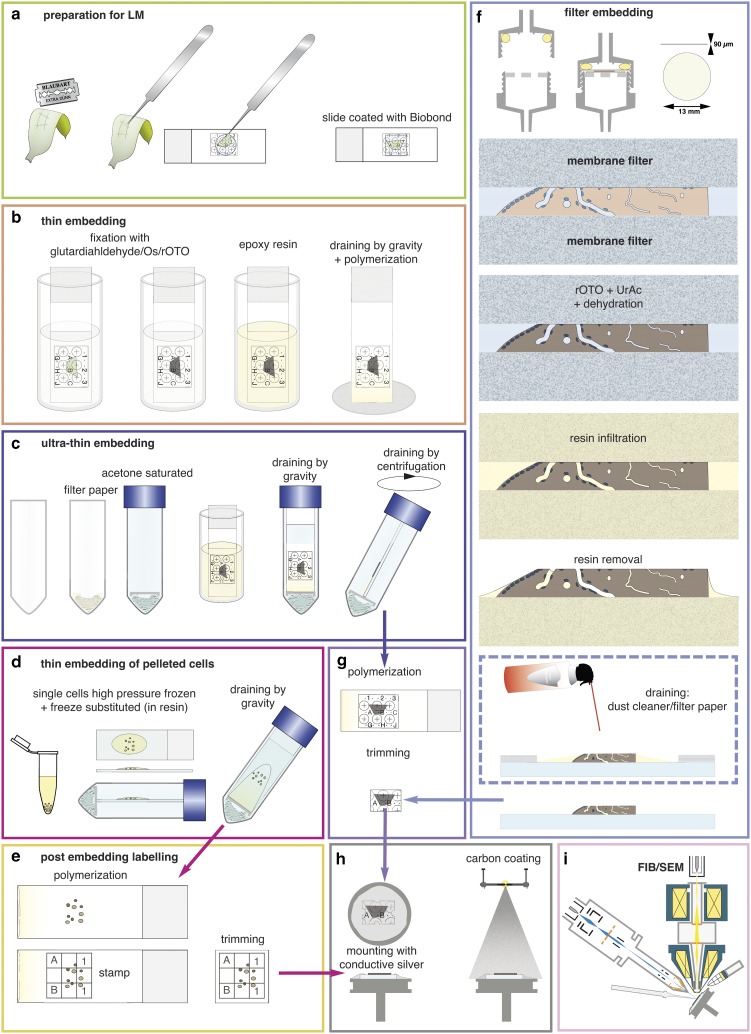
Preparations for flat embedding. If cells or tissues are either adherent or can be immobilized onto slides (**a**), fixation, post-fixation, dehydration, and infiltration with resin are carried out by submerging in cuvettes (**b**). Thin resin layers are achieved by draining (gravity) (**b**). For ultra-thin embedding, infiltrated slides are transferred into an acetone-saturated chamber for draining (gravity), which is optionally followed by centrifugation (**c**). Cells available only as resin infiltrated pellets (e.g. high pressure frozen) can be dropped onto slides and spread/drained in an acetone chamber (**d**). If resin layers obscure the coordinates of the slides, a stamp can be used for post-embedding labeling for correlative light and electron microscopy (**e**). Delicate biological samples can be infiltrated within a filter system (**f**): after light microscopy, samples are placed between acetone resistant filter membranes, sealed in a holder, processed until infiltration by flow through with a syringe. For thin embedding, samples are transferred to a glass slide (for additional LM after polymerization) before draining with filter paper or optional blowing with a dust cleaner for removal of excessive resin (**f**). For reduction of potential charging effects, specimens are trimmed to a proper size (**g**), mounted onto aluminum stubs with conductive silver by contacting the glass surface broadly. Specimens are coated with carbon by evaporation (**h**) and transferred to the focused ion beam (FIB)/scanning electron microscopy (SEM) (**i**).

Slides were coated according to the manufacturer’s instructions and stored for weeks. For a first test, a drop of sample (cyanobacteria, diatoms, green algae, filamentous algae, biofilm) was placed on a coated slide, covered with a cover slip, and gently pressed, resulting in a thin layer of cells and providing contact with the surface of the slide for sufficient adhesion (Fig. 3a). The coverslip was removed after a few minutes and the slide was gently agitated in the buffer, medium etc. and again covered by a cover slip.

#### Cell-Tak^TM^ (Corning^TM^, New York, NY, USA)

Cell-Tak^TM^ a mussel protein (Waite & Tanzer, 1981) was used for adhesion of vibratome sections of mouse brain and histological sections with a thickness up to 100 *µ*m. Cell-Tak^TM^ was applied to slides according to the manufacturer’s instructions. The cells/tissues were placed on coated slides in the buffer, covered with a covered slip gently pressed for a proper contact of the tissue to the slide.

#### Bondic® (VIKU UG, Munich, Germany)

Bondic® is a viscous adhesive polymerized by UV radiation. It is waterproof and heat resistant. A drop was spread over a slide with a coverslip. Pieces of biological material were placed in the buffer on the coated area, covered with a coverslip and exposed to UV light from the backside of the slide for a few seconds.

### Immobilization and LM Procedure

Cells/tissues were immobilized with suitable adhesives previously described. Samples were sealed with a coverslip and Fixogum (Marabu GmbH & Co. KG, Tamm, Germany) in the buffer, to prevent drying during LM imaging. ROIs were marked on a template copy with the same coordinates as on the slides. For standard CLEM documentation of ROIs, two to three different magnifications (objective: 5×, 10×, 40×) were sufficient for retrieval in SEM. Depending on specimen properties, bright field (BF), phase contrast (Ph), differential interference contrast (DIC), and epi-fluorescence or laser scanning microscopy (LSM) was used (Carl Zeiss, Oberkochen, Germany). Different areas were routinely documented, since (i) there can be a loss of few cells and/or damage during handling; (ii) some cells may show insufficient fixation quality or contrast in SEM among neighboring cells that are adequate in structure and contrast. LM can be repeated after thin embedding in epoxy resin, to monitor structural changes, control of contrast enhancement, and to check preservation of selected ROIs or to select new ones.

### Post-Fixation by Submerging

Samples were processed, by submerging slides in buffers, fixatives, solvents, and resins, in standard staining cuvettes (Fig. 3b). The customized rOTO-protocol, was based on (Willingham & Rutherford, 1984), with 1% OsO_4_ (Science Services, Munich, Germany) and 1% K_4_Fe(CN)_6_ (Merck) in cacodylate buffer for 30 min, washed three times in *aqua bidest.*, incubated with 1% thiocarbohydrazide (Merck) in *aqua bidest.* for 30 min, washed with *aqua bidest.* three times, followed by post-fixation with 1% OsO_4_ in *aqua bidest.* for 30 min. The samples were rinsed three times with *aqua bidest.* and dehydrated in a graded series of acetone (10, 20, 40, 60, 80, 100%), with a 1% uranyl acetate (Science Services) step in 20% acetone for 30 min.

### CPD

Dehydrated samples were CPD (Polaron, Montreal, Canada). After CPD, slides were either stored in a desiccator (to prevent hygroscopic water uptake and consequent ultrastructural changes) or in little transport boxes sealed with Parafilm (Merck).

### Flat Embedding in Thin and Ultra-Thin Epoxy Layers

Immobilized cells and tissues were processed and embedded on a glass slide.

#### Thin Embedding

Immobilized cells/tissues were infiltrated with 1:1 Hard-Plus Resin-812 (Science Services) in acetone for 15 min, 2:1 for 30 min, 75–100% Hard-Plus Resin-812 for 30 min at RT. The excessive resin was removed by draining (Fig. 3b).

#### Ultra-Thin Embedding

Immobilized cells/tissues were infiltrated with 1:1 Hard-Plus Resin-812 in acetone for 15 min, 2:1 (resin in acetone) for 30 min and 75–100% Hard-Plus Resin-812 for 30 min. The slides were immediately placed in a falcon tube, saturated with acetone, allowing the excessive resin to drain into filter paper at the bottom of the falcon tube for 30 min. To prevent dilution of the resin, direct contact of the slides with the filter paper, soaked in acetone, was avoided by putting a spacer (e.g. polypropylene cap) in between. As the acetone cannot volatilize, the resin concentration is not increased during infiltration and maintains its high fluidity to drain quantitatively. In some cases, when necessary, an additional centrifugation step was added (2 min; 1000 rpm). All samples were polymerized at 60 °C for 72 h (Fig. 3c).

### Protected SEM Preparation by Filter System

If tissues could not be immobilized, a filter system was used (Fig. 3f). Samples were placed, with a drop of buffer on hydrophilic, acetone-resistant 13 mm ipPore Track Etched Membranes (it4ip, Belgium), with a thickness of 12 *µ*m, a pore size of 0.4 *µ*m and a pore-density of 1×10^8^/cm^2^ to enable a sufficient flow rate by an appropriate stability. A second membrane is then placed to cover the tissue; subsequently the “membrane sandwich” is placed on top of the planar bottom part of the filter holder. The filter system is closed with the upper part of the holder, containing an O-ring (Fig. 3f). Care has to be taken that the O-ring is not contacting the “membrane sandwich” by screwing the plug. Reagents for post-fixation, dehydration, and resin infiltration were injected by 5 ml syringes (B. Braun Melsungen AG, Germany). Before polymerization, samples were transferred to slides. Draining and blotting of the excessive resin, with filter/lens paper or with a dust cleaner and monitored with a stereo lens (Fig. 3f), was effective in removing excessive resin until the samples appeared completely dry.

### Thick Epoxy Sections

Classic TEM resin blocks were trimmed and sections with a thickness of 5–10 *µ*m (depending on the cell size and density) were cut with a glass knife, then placed onto a drop of *aqua bidest.* on a laser-marked slide. If resins are brittle, the specimen can be heated with a blow dryer to ~50 °C to increase elasticity. While heating the slide to ~60–80 °C, the sections soften and stretch to their original size, which can be controlled while viewing with a stereo lens. Once the water droplet has evaporated, the sections stick to the slide.

### Mounting and Conductive Coating

Coverslips were used in their entirety, whereas glass slides were scored with a diamond scriber (Ted Pella Inc., Redding, CA, USA) and a ruler and fractured into appropriately sized pieces, to reduce the surface area of the glass and consequently potential for charging during SEM investigations (Fig. 3g). If in-lens SE or in-lens energy selective backscattered electron (EsB) detectors were needed for highest resolution, the samples were cut to smaller pieces to allow short working distances (WD) in the range of 1–2 mm. Pieces were mounted with conductive silver colloid (Plano, Wetzlar, Germany) onto standard aluminum stubs (Plano) (Fig. 3g). Specimens were sputter coated with layers of a few nanometer (3–5 nm) of platinum (Balzers AG, Liechtenstein) for high-resolution SEM at low kV (Fig. 3g). For immuno-SEM and energy dispersive X-ray (EDX) analyses, specimens were carbon coated (3–5 nm) by evaporation (Cressington Scientific Instruments UK, Waterford, UK). If specimens were prepared for FIB/SEM-tomography, a carbon coating of 10–20 nm was used for both conductivity and protection, forming a glass-like, very stable conductive layer which is still transparent for higher energetic BSEs. For high-resolution SEM at low kV (0.8–1.8 kV), slides were carbon coated before cells/tissues were grown or immobilized, to enable conductivity without any coating (Table 1). The thickness of the glass slides (approx. 1 mm) or coverslips (precision coverslips: 0.17 mm) has no influence on SEM imaging if well grounded with silver colloid.

**Table 1:** Options for Coatings and Scanning Electron Microscopy (SEM) Operation.

	Pt-sputter Coating	Carbon Coating	Carbon Coated Slide	Pt-Deposition	kV	Detector
High-resolution SEM	3–5 nm				0.8–1	SE/in-lens SE
High res. immuno-SEM (10 nm gold)		3–5 nm	(+)		1–2 5–20	EsB QBSD
High res. immuno-SEM (5 nm gold)			+		1 5–20	EsB QBSD
SEM for depth information		3–5 nm			5–30	QBSD
SEM for element analysis		3–15 nm			1–30	EDX
CPD+FIB/SEM		3–15 nm		(0.5–1 *µ*m)	1.5	EsB
Flat embedded+FIB/SEM		10–30 nm		(0.5–1 *µ*m)	1.5	EsB

SE, secondary electrons; EsB, energy selective backscattered electron; QBSD, quadrant backscatter electron detector; EDX, energy dispersive X-ray; CPD, critical point dried; FIB, focused ion beam.

### High-Resolution SEM

Samples were imaged with a Zeiss Auriga 40 FIB/SEM workstation operating under SmartSEM (Carl Zeiss Microscopy GmbH, Oberkochen, Germany). For re-localization and a rough correlation of LM images with SEM micrographs, the WD has to be large (10 mm) and the kV high (5 kV) to ensure a sufficiently low magnification with acceptable low geometrical distortion. Although surface details are best monitored at 1 kV with the In-lens SE detector, correlation with LM micrographs sometimes need as much depth information as possible, gathered by the EsB detector (at 1–5 kV) or with the 4-quadrant backscatter electron detector (QBSD) at higher accelerating voltages (5–30 kV). Thin layers of resin then become transparent and laser marks are clearly visible. Using BSE signals, a larger aperture (60 *µ*m) is necessary for a sufficient signal to noise ratio, which does not influence resolution at low and moderate kV. The high current mode (a feature of some Zeiss SEMs) is of benefit if the depth of focus is of importance: high current mode increases the active probe current by a stronger activation of the condenser lens. The resulting smaller angle of convergence increases the depth of field, which is important when imaging entire cells (with a height of 10–20 *µ*m), which is not common with SEM in standard configuration.

### High-Resolution FIB/SEM

Cells and tissues were milled and imaged with an Auriga 40 FIB/SEM workstation operating under SmartSEM or Atlas-3D (Fibics Incorporated, Ottawa, ON, Canada). Ion beam currents of 50 pA–10 nA (depending on the stability of the resin) were used. FIB/SEM milling started right in front of the target structure. Depending on the desired resolution image, voxel sizes between 2 nm and 10 nm in *x*/*y* were chosen. A milling rate that yield 2 nm slices allows the adjustment of the *z* resolution in 2 nm steps at any time during the FIB/SEM run. Due to metallic rOTO impregnation of the tissue, conduction with colloidal silver, carbon coating by evaporation, and an optional Pt-deposition upon the ROI, charging was completely avoided. As rOTO impregnation provides a strong material contrast, short exposure times down to 17 sec/image (3072×2048 pixel) could be achieved. The surface of the glass slide, as part of the image, serves as a reference for the *xz.* plane. For additional alignment in *yz*, reference lines were milled with a 50-pA beam next to the target region.

### 3D-Reconstruction

The resulting data sets were aligned using Amira^TM^ (Thermo Fisher Scientific, Waltham, MA, USA), first automatically with the module “align slices” and corrected with the “shear” function. The quality of the alignment has to be verified by the references, the slide (*xz*) and the added reference lines (*yz*) and, if necessary, corrected manually. Image stacks were segmented and reconstructed in Amira^TM^ (Thermo Fisher Scientific) and/or processed with a volume-rendering algorithm (*volren*) for direct visualization. 3D reconstructions/correlations were performed with Amira^TM^.

## Results

### Linking Samples to Coordinates

**Figure 4:**
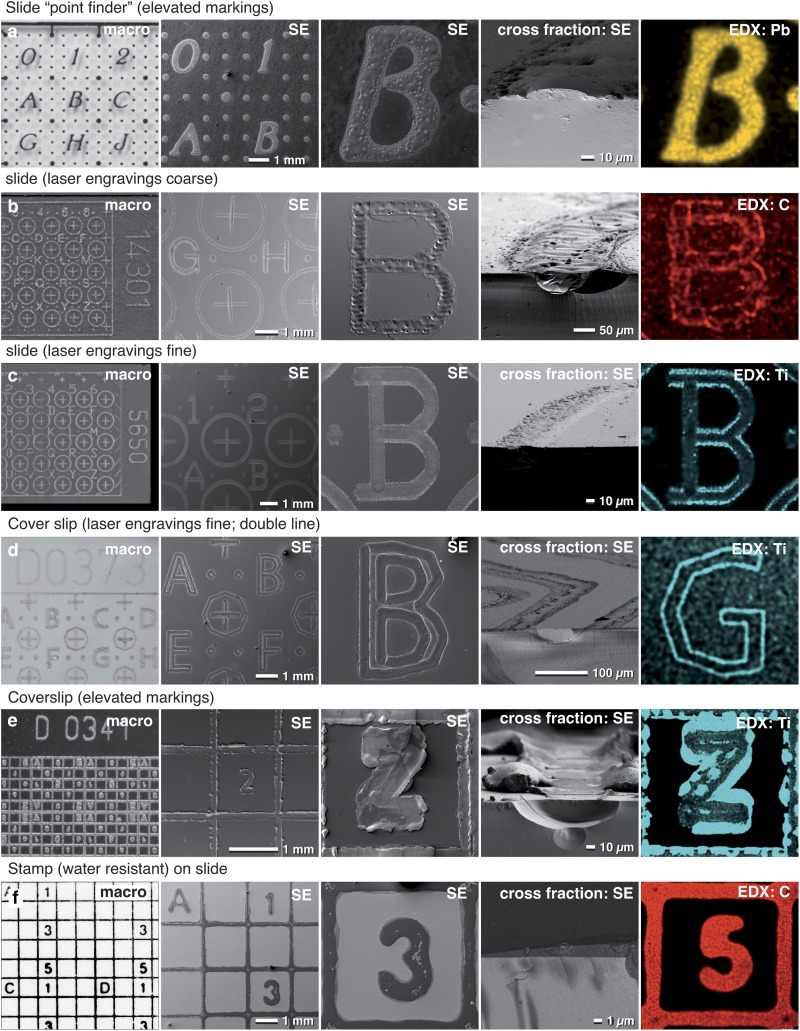
Properties of customized slides and cover slips. Macro images, light and scanning electron micrographs of slides and coverslips customized for correlative light microscopy (LM), high-resolution scanning electron microscopy (SEM) and focused ion beam (FIB)/SEM. Coordinates for slides and coverslips are either elevated (**a** and **e**), engraved (**b**) or a combination of both (**c**, **d**), suitable for different embedding properties (ultra-thin, medium-thin, thin). The coordinate system can be visualized with topography contrast and material contrast due to the sintered material. X-ray analysis can be used for visualizing of labels if they are hidden in the thin resin layer and/or for mapping of the elements characteristic for the labels (**a**). As titanium oxide mixed with organic carbon compounds is used to focus the laser (**b**–**e**), labels are visible based on their specific X-ray energies, which is beneficial for analytics or correlative light and electron microscopy (CLEM). If coordinates are printed with a stamp, the carbon-based ink is visible in black in LM, dark in SEM and gives a strong C signal with energy dispersive X-ray (EDX) (**f**).

We first tested several methods to label coordinates on glass slides and coverslips. All these labels were clearly visible in LM optical modes (bright field, DIC, phase contrast) as well as in SEM due to their topographic contrast (Fig. 4). The labels of the classic point finder (a sintered decalcomania with silk-screen printing), with a height of ~10–15 *µ*m, yielded the strongest BSE signal, due to the lead content of the paint (Fig. 4a). Depending on the downstream applications, the composition of this paint can be modified if desired.

We found that laser engravings generated much finer lines. Upon closer inspection of fractured slides, the label appeared as a slight engraving outlined by an elevated border, deriving from either sintered TiO_2_, a carbon compound, or both, which were used for focusing the laser and for the energy transfer (Figs. 4b–4e). In this regard, the term engraving is a bit misleading. For CLEM and FIB/SEM of a thin embedded specimen, elevated labels were required to poke out of the thin layer of resin (Figs. 4a, 4e). In general, when an inverted microscope was used for LSM, laser marked coverslips were preferable (Figs. 4d, 4e).

Although the fabrication of fine coordinates is limited in terms of line width and accuracy, the quality of the labeling is, in practice, of secondary importance. In fact, the unique edges of imperfect markings or engravings can be used to facilitate fast correlation. The “mesh size” of the labeling can be adjusted depending on the size of the samples, but in all cases, the coverage of the coordinates is below 15%. For samples already flat embedded on a slide or cover slip, or in situations where the labels were no longer visible due to the formation of a surface layer or hidden by epoxy resin, we used a water-resistant stamp to print a customized coordinate system on top of the specimen (Figs. 4 and 3f). After documentation and carbon coating for conductivity, the coordinates of the stamp were still visible in the SE-image by topographic and material contrast, which enabled a re-localization of the previously selected ROIs (Fig. 4f).

### Sample Immobilization

Proper immobilization is crucial to link the biological samples to the coordinate system of the substrate. This connection has to be rather strong to withstand the manipulations and buffers used during processing for SEM and FIB/SEM microscopy. Depending on the size, shape, and adhesive properties of the sample, a variety of “glues” can be applied for immobilization. For example, laser marked slides coated with poly-L-lysine, are routinely used for drop-cryo fixations of prokaryotic and eukaryotic microorganisms. Typically, these cells are abundant, so even the loss of a high percentage of cells is mostly inconsequential.

As a general rule, the larger the cells, the lower the yield of stably fixed cells remaining after sample preparation. As a result, stronger glues are required for such larger samples. In this regard, Biobond showed a rather strong adhesion to cells in addition to being easy to handle and moderate in price. Biobond was successfully used to immobilize a wide range of mobile algae. In contrast, filamentous cyanobacteria forming slime sheets were initially improperly immobilized with Biobond. However, after choosing strains which do not form sheets, these cells were successfully immobilized.

Next, Cell-Tak^TM^, derived from a mussel protein, is a very strong adhesive. Cell-Tak^TM^ showed stronger adhesion properties than Biobond, which were beneficial for fixing larger tissue sections to slides. However, immobilizing thin or fragile tissue sections over their entire surface, without any folds, can be difficult. As soon as the tissue sections are in contact with the mussel protein, they will immediately adhere. If any folds occur due unevenly laying the tissue on the slide, the tissue cannot be flattened without disruption. As Cell-Tak^TM^ has to be applied for each specimen individually, the adhesive layers vary in thickness and are not as thin and uniform compared with Biobond coating. This may be a disadvantage when investigating samples such as cellular protrusions, flagella, or cilia. These structures would be difficult to distinguish at higher magnification due to the uneven topography of the glue.

Bondic®, developed as an adhesive, can be used to immobilize samples in aqueous conditions. This adhesive is polymerized by radiating the contact area for a few seconds with UV light from the backside of the slide. It is an inexpensive and strong adhesive. However, the layers are rather thick and therefore only suitable for large and sturdy samples, such as those that can be handled with forceps.

For flat embedding of delicate and fragile tissues sections, we developed a customized filter system for tissue sections, which mechanically stabilizes the sample. To this end, the tissue sections are placed between two filter membranes and screwed in a filter holder, thereby ensuring that no significant forces perturb the geometry of the sections during the entire fixation and embedding process (Fig. 3f). In addition, only small amounts of post-fixation solutions are needed, due to the small volume of the holder. Importantly, large vibratome sections remain flat, even during dehydration, as they are mechanically stabilized, which is essential for FIB/SEM. Finally, excess resin is then removed, and samples are polymerized on laser marked slides (if coordinates are needed). A variety of samples are quite suitable for processing using the customized filter system:

∙ large histological sections;
∙ samples, which cannot be immobilized by adhesives;
∙ samples, which are very fragile; and
∙ samples, which have to remain flat during preparation/dehydration.

## Choosing the Right Embedding

**Figure 5:**
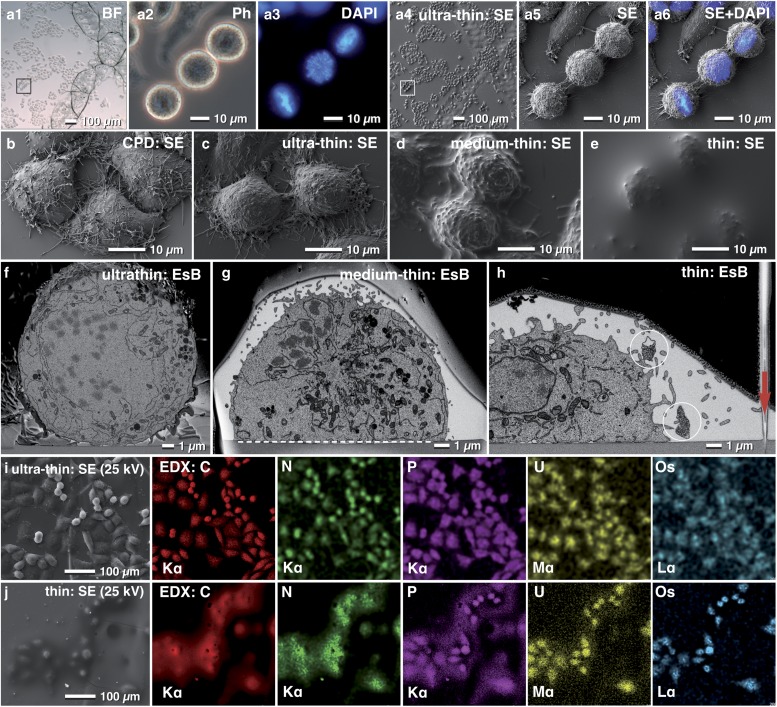
Re-localization after flat embedding. Light micrographs of HeLa cells grown on a laser-marked slide to the left to the letter X (**a1**). After 4′,6-Diamidin-2-phenylindol (DAPI) staining, metaphase cells were selected (**a2**, **a3**) and re-localized in SEM, already visible in the overview (**a4**, **a5**). DAPI and secondary electron (SE) image were superimposed to define a precise milling frame (**a6**). Scanning electron micrographs of critical point dried HeLa cells, compared with cells, embedded with different thick layers of resin. The topographic details of critical point dried cells (**c**) were preserved after ultra-thin embedding (**c**). Structural details like filopodia were clearly visible, whereas, after medium-thin embedding, they were obscured (**d**). Thin embedded cells, covered by a few microns of resin, could be still re-located and identified as small humps (**e**). Comparison of cross sections of HeLa cells embedded ultra-thin (**f**), medium-thin (**g**) and thin (**h**) in epoxy resin. The thicker the covering resin layer, the less topographic details of the cell surface were visible, however, the risk of curtaining is reduced by the smooth surface (**h**). The glass slide served as an absolute reference plane (xz) for alignment (**g**; dashed line). Lines, milled parallel to the regions of interest (ROI) into the slide in *xz* direction (**h**; arrow), served as a third reference plane for precise alignment of the image stack in three dimension. The X-ray signals were used for verification of resin thickness. The carbon signal of ultra-thin embedded cells, clearly visible at 25 kV (**i**), became blurred after thin embedding, due to the carbon portion of the covering resin (**j**). P, U and Os mapping of thin embedded cells revealed only a section of the cells appearing “in focus” as the higher energetic K-line of phosphorus, the M-line of uranium and the L-line of Os osmium were excited only from higher energetic electrons near the surface (Fig. 5j).

Depending on the scientific question, it is often desirable to control the thickness of the resin layer for flat embedding. In this regard, we demonstrate adjusting the embedding thickness with HeLa cells (Fig. 5). With the portfolio of procedures ranging from simply draining the resin, to centrifugation and infiltration in an acetone-saturated chamber, the thickness of the resin layer can be adapted from ultra-thin to thin embedding. These procedures were suitable for a broad spectrum of adherent or immobilized cells and tissues (Figs. 5b–5e). In general, a thin resin layer was advantageous for the stability of the FIB-SEM run (Fig. 5h). In contrast, ultra-thin embedding was preferable for CLEM of inner cellular features (e.g. centrosomes, kinetochore), which had to be re-localized with high precision in FIB/SEM (Figs. 5c, 5f). An efficient way of producing very thin layers of resin consists of embedding the specimens in 75–100% (resin/acetone). Since samples are infiltrated in a saturated acetone atmosphere (Fig. 3c), the resin concentration is not increased and maintains its high fluidity. After an appropriate time of infiltration, the excess resin/acetone mixture is drained either by gravity or centrifugation, resulting in an extremely thin resin layer after polymerization (Figs. 3c, 5a, 5c, 5f, 5i). Minor milling artifacts at the surface, which are the result of an extensively textured cellular surface, can be ignored, if the target area is located within the cell and if these artifacts do not influence the ROI itself (Fig. 5f). For correlative LM and SEM or FIB/SEM, ultra-thin to medium-thin embedding is beneficial, since SEM and LM images (DIC, bright field) were almost identical qualitatively and their correlation was very precise (Fig. 5a), comparable with CPD cells (compare: Fig. 5b with 5c), but with a substantial gain in resolution using the EsB signal, and visibly less curtaining during milling (Figs. 5f, 5g). If thin embedding is desired, the correlation is impeded because cells are partially hidden within resin droplets (Fig. 5h). Nevertheless, the identification of selected cells was still carried out by superimposing LM and SEM micrographs. For experiments relying on cellular surface information or looking at externalized vesicles such as exosomes, thin embedding is preferable (Fig. 5h). In all cases, regardless of the resin thickness, references (glass slide and reference lines) were clearly visible in each tomographic image (Fig. 5h).

X-ray analysis allows the estimation of resin thickness (before FIB/SEM) by increasing the kV until the silicon signal, derived from the glass slide becomes prominent (Figs. 5i, 5j). The X-ray signals of carbon, nitrogen, phosphorus, osmium, and uranium of ultra-thin and thin embedded HeLa cells at 25 kV correlated with the outlines of nearly all cells (SE image) present in the mapping area. By passing through the covering resin layer of thin embedded cells (in contrast to ultra-thin embedded cells), the primary electron beam loses a lot of energy and spreads in diameter, resulting in a blurry appearance of the cells (Fig. 5j).

Care must be taken during SEM and preparation for FIB-milling: thin resin layers on glass slides are sometimes very sensitive to the electron beam. Higher magnifications, used for focusing and correction of astigmatism, rapidly lead to the formation of “bubbles” in the resin, which look striking, but did not influence further milling. Low magnifications and using an appropriate kV are recommended for SEM and FIB adjustment. Once the FIB/SEM milling process has started, the energy input is concentrated onto the block face, and the risk of “bubbles” is negligible. For thin specimens (1–5 *µ*m) a very low ion beam current (50 pA, as used for FIB imaging) is recommended.

## Examples for Analytical SEM and FIB/SEM and Possibilities for CLEM

### Chromosomes: Immuno-Labeling—from LM to FIB/SEM

**Figure 6:**
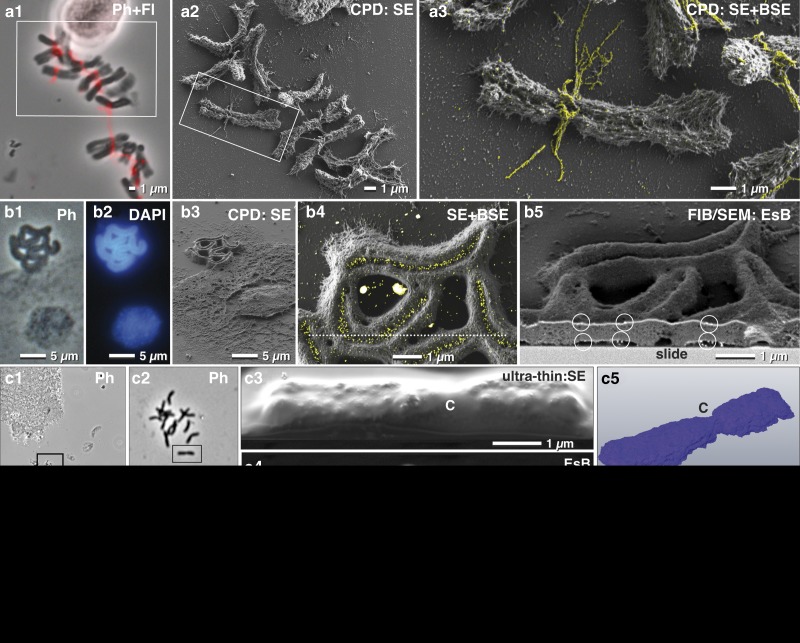
Correlative light and electron microscopy of isolated chromosomes. High-resolution correlative light microscopy, scanning electron microscopy and focused ion beam/SEM of specifically labeled spelt metaphase chromosomes, fixed onto carbon-coated slides. Chromosomes, immuno-labeled for *α*-tubulin, were selected with phase contrast combined with fluorescence (**a1**), re-located in SEM (**a2**) with secondary electrons (SE) and analyzed with both, SE- and backscattered electron (BSE)-signal for detection of FluoroNanogold^™^ labels enhanced with silver (yellow) located at bundles of tubulin attached to the centromere (**a3**; merging of SE-image with the colored BSE image). Holocentric chromosomes of *Luzula elegans* were visualized with phase contrast (**b1**), and fluorescence of DAPI (**b2**), re-located in SEM (**b3**). The gold-labeled antibodies against a centromere-specific phosphorylated histone (anti-Histone-H2A) were localized within the centromeric groove (**b4**; merged SE-image and BSE image). For verification of label distribution, the specimen was carbon coated before FIB/SEM milling. Gold-labeled antibodies were detected on both sides of the chromosomes, predominantly located at the surface (**b5**; framed area). Barley metaphase chromosomes were fixed onto slides (**c1**; **c2**=framed area of **c1**) stained for DNA distribution with platinum blue and embedded in the water-soluble resin Moviol (**c3**; SE-image) to prevent shrinkage during dehydration in ethanol/acetone. FIB/SEM tomography (**c4**=BSE) shows the global Pt (DNA) distribution in three dimension (**c5**). C=centromere. A migrating human platelet collecting fibrin-trapped *E. coli* (**d1**) (orange=tdTomato; green=fibrin-Alexa-488-10 nm-gold). SEM micrograph of the platelet of (**d1**) after thin embedding and platinum deposition (**d2**). FIB-SEM section shows *E. coli* accumulating at the surface of the platelets (**d3**). A 3D-rendered FIB-SEM stack of the same platelet (**d4**) demonstrates the accumulation of the *E. coli* (orange) via fibrin (immuno-gold labeled=yellow).

The topography of isolated cell organelles or single cells can be investigated with high resolution, after application of a thin metal coating (3–5 nm platinum), using a short WD (1–2 mm) and with the in-lens SE detector at 0.8–1.2 kV. However, the platinum coating is incompatible with metal-based staining such as platinum blue for DNA or immuno-labeling with small gold colloids (5 nm). Even the thinnest platinum coating will prohibit detection of metal-based staining or gold-labels with the EsB-detector (Figs. 6a3, 6b4). Carbon coating can be used for conductive coating if higher currents are required, but with a dramatic loss of high-resolution topographic information gathered at 1 kV. However, for a high-resolution analytical investigation of isolated biological structures such as chromosomes, carbon coating of the substrate (glass slides) before applying the sample is ideal since: (i) the problem of charging can be minimized and even eliminated, either at low kV or due to the conductive carbon layer; and (ii) the rather dark background in the BSE image enables high-resolution SE and high contrast BSE imaging at very low kV (0.8–1.5 kV).

Carbon-coated slides remain translucent after coating, therefore enabling LM to select ROIs beforehand. Since the re-localization of small biological structures such as mitotic chromosomes is not trivial, a linked reference coordinate system is critical. For example, the mitotic index for spelt is very low (2–15%), and therefore a previous selection in of ROIs in LM was essential (Fig. 6a1). FluoroNanogold^TM^-labeled antibodies against alpha-tubulin were located at fibrillar bundles at the centromeric region of the mitotic chromosomes (Figs. 6a2, 6a3). Analytical investigations of metal impregnations at different depths of the sample were carried out by varying the negative grid voltage of the in-lens energy selective BSE detector for high resolution (Fig. 6).

To detect labeled structures located inside a biological structure, the kV must usually be increased. Typically, charging begins e.g. at 2–3 kV, but disappears at higher kV as the electrons penetrate the specimen and reach the conductive carbon layer. In this case, the system must be operated at two different accelerating voltages: 1 kV to image the surface topography and surface-located labels (in-lens SE detector, EsB detector) (Fig. 6a2, 6a3) and 10 kV for high-resolution imaging of labels in the interior of the sample (QBSD). Additionally, the low BSE-yield of carbon prevents interfering BSE signals from the substrate. To demonstrate this, holocentric chromosomes of *Luzula elegans* were stained with DAPI (for DNA) and with gold-labeled antibodies against a phosphorylated variant of Histone H2A (for centromeres). These chromosomes were first visualized with phase contrast (Fig. 6b1), and with fluorescence microscopy (Fig. 6b2) and then re-located in SEM (Fig. 6b3). Gold-labeled antibodies (anti-Histone-H2A) were then visibly localized within the centromeric groove (Fig. 6b4). To verify the spatial distribution of the gold labels, the specimen was carbon coated before FIB/SEM milling, to avoid charging during milling. As a result, gold labels were clearly visible on both sides of the chromosomes (Fig. 6b5).

CPD samples of chromosomes can also be used to study DNA distribution with FIB/SEM. However, CPD may cause shrinkage and formation of cavities. To avoid dehydration artifacts, embedding in Moviol, a water-soluble resin, is beneficial, and offers a smooth block face which yields the highest resolution (Fig. 6c). The BSE signal of the Pt-stained chromosomes showed the global distribution of the DNA (Figs. 6c4, 6c5).

For correlative LM and FIB/SEM microscopy, samples are commonly embedded in a resin block together with the substrate. The substrate is then removed after polymerization by thermal shock, thereby leaving the cells in the resin. However, if the experiment involves examining how the cell contacts the substrate, for example, to analyze attachment or migrating activity, then the substrate cannot be removed. Therefore, in such cases, ultra-thin or thin embedding directly on the slide is required. With immuno-fluorescence microscopy, it was demonstrated that human platelets migrate and pile up on the adhesive substrate together with any bound particulate material. This occurs when actomyosin-dependent traction forces overcome substrate resistance (Gaertner et al., 2017) (Fig. 6d1). Once removed, fibrinogen is transported toward the center (pseudonucleus) of migrating platelets, remaining on the platelet surface, mainly within invaginations of the open canalicular system. After LM (Fig. 6d1) platelets were thin embedded, re-located in SEM and covered with two platinum protection layers by ion beam deposition (Fig. 6d2). Additionally, a carbon layer was implemented between these two layers for milling high contrast tracking lines for FIB/SEM microscopy using Atlas3D hard- and software. These tracking lines enabled correction of astigmatism and focus (*autostig and autofocus*) during the FIB/SEM run (Fig. 6d3). *Escherichia coli* cells were collected and accumulated at the pseudonucleus together with the fibrin(ogen)-forming bundles of several bacteria (Fig. 6d3). A 3D-rendered FIB-SEM stack of the platelets demonstrates the accumulation of the *E. coli* via fibrin (Fig. 6d4).

### Microorganisms: From fast Results To High Resolution

**Figure 7:**
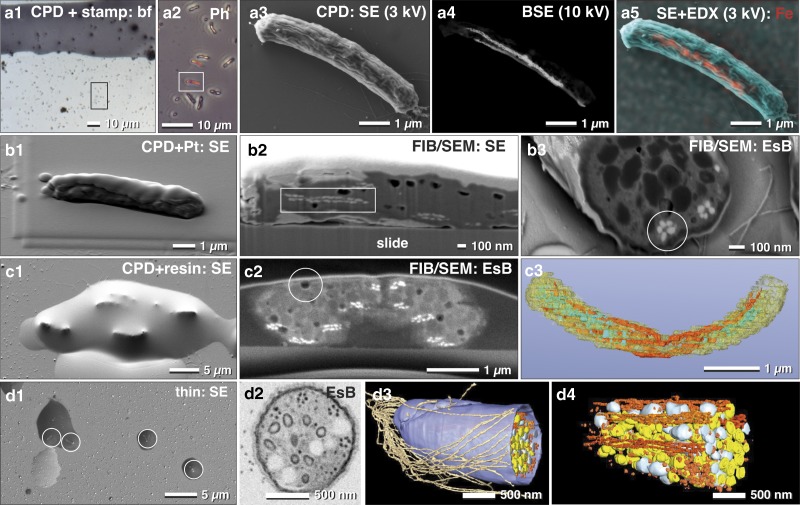
Correlative light and electron microscopy of immobilized and pelleted prokaryotic cells. Light and scanning electron micrographs of *Magnetobacterium bavaricum* cells, within a mixed-culture prepared for correlative light microscopy and focused ion beam (FIB)/scanning electron microscopy (SEM). A slide with critical point dried bacteria was labeled with a water-resistant stamp for reference coordinates (**a1**). A target cell of was documented by phase contrast (**a2**; framed area) and re-localized in SEM with secondary electrons (SE) at low kV (**a3**) and with backscattered electrons (BSE) at higher kV, for detection of the magnetosomes by material contrast (**a4**). Energy dispersive X-ray analysis mapping of iron distribution (L-line of Fe at 3 kV) was superimposed to the SE image, confirming the characteristic Fe component of magnetosomes (**a5**; red). After coating the cells with platinum by ion beam deposition (**b1**) and FIB/SEM milling, in both SE (**b2**) and BSE images (**b3**) the magnetosome chains were clearly visible by material contrast, longitudinally (**b2**; rectangle) or cross-sectioned (**b3**; circle). For better milling properties, critical point dried cells were flat embedded in epoxy resin (**c1**) and milled again with significantly better image quality (**c2**). Besides the magnetosome chains, storage granules (poly-*β*-hydroxy butyrate (PHB) and sulfur) were slightly distinguishable (**c2**). Embedded cells are still beam sensitive: little holes formed during milling, even at low ion-currents (**c2**; circle). A rough 3D visualization was achieved in a short time using the threshold tool (**c3**). Best results were obtained if a pellet of high pressure frozen and freeze substituted magnetobateria is spread and embedded on slides in small droplets of resin (**d1**) for FIB/SEM-tomography (**d2**). Several structural details were reconstructed with high resolution in 3D (**d3**; **d4**) such as the number and arrangement of the magnetosomes and their chains, storage granules (sulfur=yellow; PHB=white), cellular envelope (blue) and flagella (brown). EsB, energy selective backscattered electron.

For non-adherent microorganisms, proper immobilization is a critical requirement for cost-effective FIB/SEM high-resolution experiments. In this regard, poly-L-lysine coated slides are widely known to provide adequate adhesion of prokaryotic cells. In order to demonstrate the potential and widespread applicability of fast, analytical investigations with FIB/SEM, we examined a mixed culture, containing magnetobacteria. While EDX in SEM is not commonly used in biology, it remains a very effective way to reveal for, example, metallic inclusions etc. Typically, only a minor fraction of *M. bavaricum* is found within an enriched mixed culture. Cells, immobilized by drop-cryo fixation, were CPD. ROIs containing magnetobacteria were then selected in LM by their characteristic size and shape (Figs. 7a1, 7a2). In order to retrieve the selected ROIs in SEM, we applied a stamp with a reference coordinate system on top of the CPD sample (Fig. 7a3). Chains of magnetosomes were then visualized by BSE at higher voltages (Fig. 7a4) and EDX mapping clearly confirmed and highlighted their iron content (Fig. 7a5). Together, these data allow statistical quantification of the number of magnetosome chains, their length, and their position.

There are, however, some limitations to using CPD specimens immobilized onto glass slides for EDX analysis, due to the strong signal from the glass (Si, O, and their numerous additional components). However, if beam damage is not a severe problem and if the elements have their energy lines in a suitable range, element analysis can be performed at low kV (1–3 kV) (Fig. 7a5).

We then obtained the three-dimensional distribution and architecture of single magnetosomes with FIB/SEM (Fig. 7b). CPD samples were post embedded with resin for higher resolution, compared with non-embedded samples (compare: Fig. 7b2 with 7c2). The cellular matrix was filled with epoxy resin, with resulted in a smoother block face. Since the topographic contrast of BSE is reduced, the signal to noise ratio of BSE is significantly improved (Fig. 7c2). We obtained the best ultra-structural preservation and resolution of structural details when magnetobacteria were high pressure frozen and freeze-substituted (as described by Jogler et al., 2011), then infiltrated in resin and spread on glass slides before polymerization (Fig. 7d1). Single cells, located in droplets of resin, still offer the possibility for post-embedding correlation with LM to identify target cells and enable directed (longitudinally or cross-sectioned) milling for efficient FIB/SEM microscopy (Fig. 7d2). Finally, structural details were reconstructed with high resolution in 3D, allowing for quantification of number and distribution of multiple cellular structures: single magnetosomes and chains, storage granules of sulfur and poly-β-hydroxybutyrate, as well as the cellular envelope and the bundle flagella (Figs. 7d3, 7d4).

### Multicellular organisms: Targeted FIB/SEM

**Figure 8:**
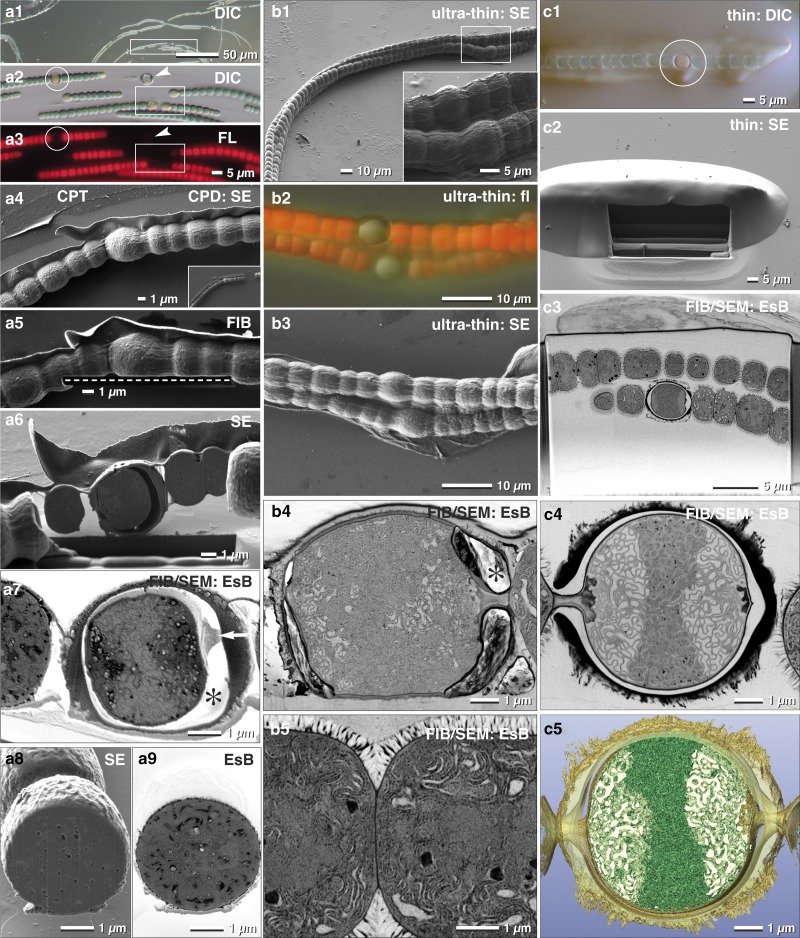
Comparison of critical point dried and embedded cells. Correlative light and electron microscopy (CLEM) of *Anabaena catenula* was facilitated by immobilization of filaments with Biobond. DIC (**a1**, **a2**) and fluorescence microscopy were used to demonstrate viability (**a3**) and re-localization in SEM (**a4**). Fine topographic details of vegetative cells and heterocysts were compared at low kV with high resolution (**a4**). Oriented focused ion beam(FIB)/scanning electron microscopy (SEM) milling of both cell types was easily achieved, as the desired milling plane could be defined with high precision in FIB mode (**a5**; dashed line). Heterocysts were stable for milling (**a6**); their connecting cytoplasmic strands to neighboring vegetative cells were clearly visible (**a7**; arrow), as well as a characteristic gap between the protoplast and the cell wall (**a7**; *) —possibly a shrinking artifact from dehydration. The cytoplasm exhibits some small holes, likely milling artifacts, best seen in SE images (**a8**), compared with the material contrast of metal impregnated cellular substructures, striking in the energy selective backscattered electron (EsB) image (**a9**). As high pressure frozen cells cannot be immobilized, they were infiltrated in resin, spread and ultra-thin embedded on laser marked slides (**b1**). Surprisingly, the autofluorescence of the vegetative cells was preserved (**b2**), although to a much weaker extent, and could be used for “post-embedding CLEM”, control of viability and classifying the developmental stage of heterocysts (**b2**). The resolution of cell topography was only slightly reduced (**b3**). Hence, every filament and individual heterocyst was re-located immediately. The FIB/SEM images have excellent resolution of the elaborate thylakoid membranes of both, the heterocysts (**b4**) and the vegetative cells (**b5**), strikingly different in their arrangement. Due to the short infiltration time and the impeded diffusion by the protective cell wall, the gap between cytoplasm and cell wall was not filled with resin (**b4**;*). For optimal FIB/SEM tomography, high-pressure frozen (HPF) filaments were embedded in thicker layers of resin. Light microscopy and SEM were used to localize the heterocysts (**c1**; circle), along with the preserved autofluorescence of chlorophyll of vegetative cells (**c1**). After milling a short ramp, the heterocyst was reached (**c2**) and high-resolution FIB/SEM stacks were collected (**c3** and **c4**) and used for three-dimensional reconstruction (**c5**).

As discussed earlier, the larger the sample, the more elaborate is its immobilization, since different surface properties often require specific adhesives. Many multicellular cyanobacteria, such as *Anabaena,* produce specialized nitrogen-fixing heterocysts, which are organized in filaments. These filaments are characterized by a regulated developmental pattern of single heterocysts, separated by vegetative cells. We found that Biobond was sufficient for proper adhesion of *Anabaena* filaments to slides. With LM (DIC, fluorescence), we selected and documented different stages of the transition from vegetative cells to heterocysts, according to the coordinates of the slides (Fig. 8a1–8a3). ROIs were then easily retrieved since the appearance of the filaments was essentially identical in SEM when compared with LM micrographs (Fig. 8a4).

FIB/SEM acquisition of CPD processed samples started directly in front of the selected heterocyst, which allowed setting a precise milling direction (in this case, longitudinally) (Fig. 8a4). Since the sample is not embedded within an epoxy resin, milling a ramp to access the target region is unnecessary. Excavations between membranes gave a strong topographical contrast (Fig. 8a5) at the expense of the BSE signal and consequentially resolution (Fig. 8a6). Of note, the large gap between the cell wall and the membrane of the heterocyst is at risk of charging. When comparing SE and BSE signals of vegetative cells, the block-face was visibly much smoother due to their dense, compact thylakoid system (Figs. 8a7, 8a8), even though, small holes still disturbed the image quality (Fig. 8a8).

Conveniently, the auto-fluorescence of *Anabaena* filaments was maintained after ultra-thin embedding and high-pressure freezing (Figs. 8b1, 8b2). This allows easy verification of the developmental status of heterocysts, and as a result, the quality of FIB/SEM was significantly improved (Figs. 8b4, 8b5). This improvement takes place even though the space between the cell wall and protoplast of the heterocysts remained empty (Fig. 8b4). We obtained the best results when thicker layers of resin were applied (Fig. 8c). A small ramp had to be milled in front of the target, however, it is clearly smaller compared with classic embedding protocols (Figs. 8c2, 8c3). As a result, single thylakoid membranes were clearly resolvable (Fig. 8c4), which then enabled striking 3D reconstructions, offering insights into the complex architecture down to 2 nm voxel sizes (Fig. 8c5).

### Tissues: From LM to FIB/SEM

**Figure 9:**
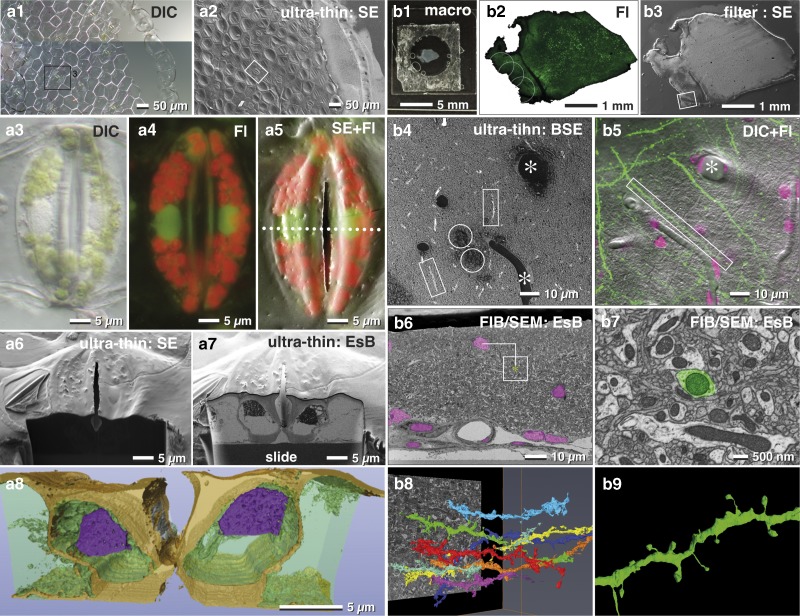
Correlative light and electron microscopy (CLEM) of thin embedded tissues. Light (LM) and scanning electron micrographs (SEM) of an isolated epidermis of *Tradescantia zebrina* used for correlative LM and focused ion beam (FIB)/SEM of oriented sectioning of stomata. The vital epidermis was immobilized to a laser marked slide with Bondic® (**a1**), fixed and ultra-thin embedded in epoxy resin for SEM (**a2**). After fixation stomata were imaged with DIC (**a3**; stomata of **a1/a2**) and fluorescence (**a4**). After flat embedding, the stomata were easily re-localized in SEM (**a2**; framed are corresponds to stomata labeled in a1). SEM and LM were superimposed to localize nuclei (**a5**). A ramp was milled with a higher ion-beam-energy (**a6**) until the target region was reached. With a lower ion beam current, serial block face images of the nuclei were acquired (**a7**) for tomographic 3D reconstruction (**a8**). Vibratome section of a mouse brain (**b1**) with GFP labeled dendrites (**b2**), processed with the filter system and flat embedded for FIB/SEM (**b3**). The target region (**b3**; framed are) was imaged at higher kV with backscattered electrons (BSE), for visualizing resin filled vessels (**b4**; *), nuclei (**b4**; circles) and axons (**b4**; rectangles), which was then correlated to LM of dendrites and nuclei (**b5**; merged images of DIC, GFP and DRAQ5). Nuclei and vessels visible in the key frames of FIB/SEM image stacks (**b6**) served for precise triangulation of the target dendrite (**b7**) and its three-dimensional reconstruction among neighboring dendrites (**b8** and b9).

Immobilizing tissue sections to a slide stabilizes the sample during SEM preparation. This, in turn, keeps the sample flat and allows thin embedding directly on the substrate, without losing orientation. In our hands, tissues were adequately immobilized with Biobond or Cell-Tak^TM^. To look further into this, we imaged a piece of *Tradescantia* epidermis, which inherited several stomata (Fig. 9a1). To determine the position of organelles such as nuclei or chloroplasts, we imaged the sample at higher magnification with different optical modes (DIC, fluorescence, Figs. 9a3, 9a4). Removal of excess resin is of primary importance. To this end, if tissues are tightly fixed (e.g. Cell-Tak^TM^), a moderate centrifugation (500 rpm for 5 min) will remove most or all of the resin obscuring the surface. After polymerization, the SEM image was similar to the LM image (Fig. 9a2), which enabled an easy and fast correlation of these two images (Fig. 9a5). This demonstrates that the target area had sufficient topographic contrast to recognize the stomata over the entire epidermis (Fig. 9a2). This, in turn, allowed for precise and directed milling (longitudinal or cross-sectioned) with FIB (Figs. 9a6, 9a7) and subsequently 3D reconstruction (Fig. 9a8).

In neuroscience, FIB/SEM became an important tool for ultrastructural studies addressing connectomics of the entire brain. For example, vibratome sections of brain tissues are commonly used to study neuronal processes (Fig. 9b1). Slices can be rather large (>1 cm^2^) or very fragile, which makes adequate immobilization onto slides particularly challenging. In this regard, the filter system described earlier facilitates a gentle post-fixation, dehydration and infiltration of resin until the sections are in a stable condition (Fig. 3f). Superimposition of LM and SEM micrographs using the characteristic outline of the brain section was generally sufficient for a rough correlation (Figs. 9b2, 9b3). Natural markers like blood vessels and nuclei, detectable in both LM and SEM images, served as fiducial markers to re-locate ROIs within the SEM (Figs. 9b4, 9b5). The distinctive shapes and sizes of structures at the surface, or sectioned natural landmarks (e.g. blood vessels appear as channels or large holes, nuclei as dark dots), facilitated a subsequent superimposition of light and electron micrographs and to define the target area in x/y direction. The depth of the target dendrite within the tissue was determined by aligning block face images with the corresponding micrographs of the LSM stacks and triangulation of the respective landmarks (Fig. 9b6). A rough 3D reconstruction of potential target dendrites within the ROI (Fig. 9b8) revealed the selected dendrite by its unique spine arrangement (Figs. 9b7, 9b9).

### Epoxy Sections for economic FIB/SEM

**Figure 10:**
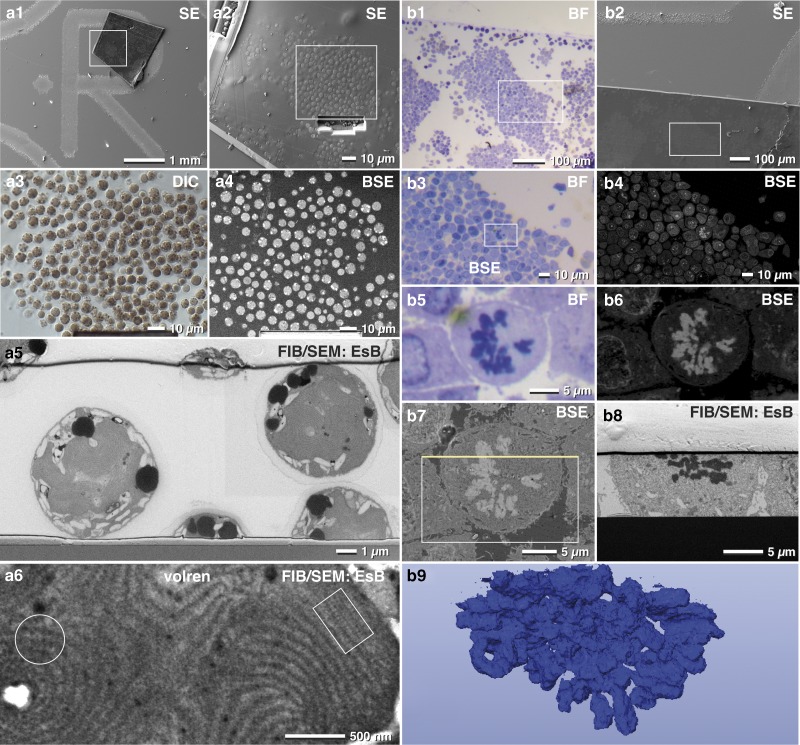
Advantages of thick resin sections for correlative light and electron microscopy. A 10-*µ*m thick microtome section of a resin embedded culture of the red algae *Porphyridium purpureum* was immobilized to onto a laser marked slide (**a1**). The cells were visible in scanning electron microscopy (SEM) by topography (**a2**) and can be compared with light microscopy (LM) (**a3**). Target cells were selected by comparing LM (**a3**) and backscattered electrons (BSE) images (**a4**). After milling a short ramp, image stacks of cells were acquired (**a5**). High resolution confirmed the arrangement of phycobilisomes in side view (**a6**; rectangle) or front view (**a6**; circle). Light and scanning electron micrographs of SKBR3 breast cancer cells fixed as a pellet and processed for conventional transmission electron microscopy. Thick sections (10 *µ*m) were mounted onto laser-marked slides and stained with toluidine blue (**b1**). The selected section were rapidly re-located in SEM (**b2**). At higher voltage, the material contrast of the SEM image (BSE signal) allowed precise localization of target cells in metaphase, imaged in LM (**b3**, **b5**) and SEM (**b4**, **b6**). After milling a short ramp, the target cell was milled (**b7**; rectangle). The yellow line marks the focused ion beam block face image of **b8**. A three-dimensional reconstruction of chromosomes was quickly achieved with the threshold tool (**b9**).

Several aspects favor the use of thick epoxy sections for FIB/SEM: (i) parts of biological specimens cannot be immobilized for various reasons and have to be embedded conventionally within resin blocks; (ii) an archive of samples already exists, which may be used for FIB/SEM years after initial preparation; (iii) samples were investigated with TEM and corresponding 3D stacks are desired of the same ROI. We found that resin sections mounted on laser marked slides was an elegant way for FIB/SEM milling of target structures embedded in resin blocks. The high potential and efficiency of thick sections was demonstrated for the red algae *Porphyridium purpureum* (Fig. 10a) and for SKBR3 breast cancer cells (Fig. 10b). ROIs were selected with bright field, DIC or phase contrast (Figs. 10b1, 10b3, 10b5). As sections are typically in the millimeter scale (feed size), the correlation was easily carried out by merging LM and SEM micrographs. At moderate kV (3–5 kV) *Porphyridium purpureum* were detected with the BSE signal (Fig. 10a4). Cells that overlapped in LM and SEM were located at the surface and already inter-sectioned. Cells visible in LM but not in SEM were intact within the section (compare Fig. 10a3 with 10a4). We then selected intact cells and directly targeted them with FIB/SEM, omitting the need for a large trench to find whole cells by chance (Figs. 10a5; 11h). Stages of mitosis, infected cells, cells undergoing apoptosis etc. were found with different optical modes (BF, Ph, DIC) (Figs. 10b1–10b5). We selected a mitotic cancer cell, then milled and imaged it with FIB/SEM and finally reconstructed it in 3D (Fig. 10b). Ten-micron sections appeared a bit more sensitive to the ion beam. It is thus recommended to reduce the ion beam energy, which is not a problem as milling speed is generally much faster than imaging time. In addition, the use of a harder mixture of epoxy resin (EMS Hard-Plus Resin 812) is recommended.

**Figure 11:**
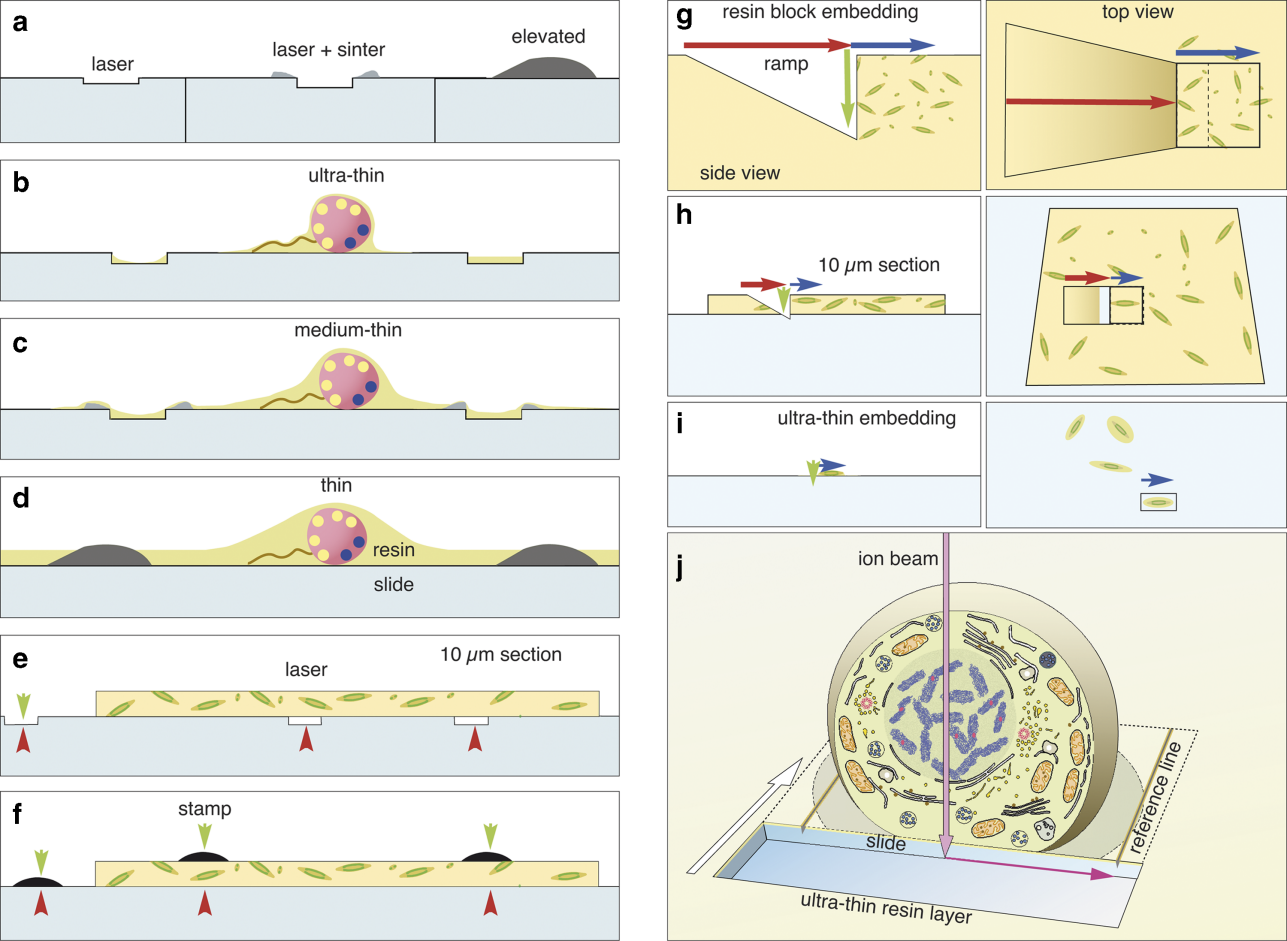
Choice of label type for correlative light and electron microscopy (CLEM). Fine customized labels can be produced with a laser (**a**). If TiO_2_ is used for focusing the laser, a sintered relief outlines the labels. Elevated labels can be produced by silk-screen printing or sintering of transfer pictures (decalcomania). Depending on the desired thickness of the resin layer, the adequate labels are used for ultra-thin embedding (**b**), thin embedding (**c**), or thicker resin layers (**d**). Thick microtome sections (5–10 *µ*m) can be mounted onto laser marked slides (**e**) or on standard slides and labeled with a stamp (**f**). Coordinates are always visible in light microscopy (red arrowheads) and in part in scanning electron microscopy (SEM) (green arrowheads) and can be used re-location directly or by triangulation. Advantage of flat samples for focused ion beam (FIB)/SEM If a single cell within a pellet (**g**) has to be reconstructed with FIB/SEM, a ramp must be milled (**g**; red arrow) to reach the desired depth (green arrow). As cells are randomly oriented, a large volume has to be sectioned (blue arrow) for an image stack to including a complete cell in the desired orientation (cross section, longitudinal section). The milling area (boxed area) has to be broader than the ROI due to re-deposition. Moderate magnifications have to be chosen to enhance the chance of catching a cell in the right orientation, thereby limiting resolution. When 10 *µ*m resin sections are used for CLEM, the volume of the ramp is significantly reduced, and the surface of the slide can be used as a reference plane (*xy*) for alignment (**h**). If cells are embedded ultra-thin (**i**), the ramp is needless and only the actual size of the regions of interest (ROI) is milled (**i**; blue arrow, boxed area). The milling area can be precisely adjusted for each cell both in size and orientation. The magnification can be adapted, from the start of milling to the size of the target cell. If an ultra-thin embedded cell is used for FIB/SEM (**j**), the block face defines the first plane for the cubic volume of the image stack. The glass slide gives a second absolute reference plane (*xz*) for alignment. Lines, milled parallel to the ROI into the slide, serve as a third reference plane for precise alignment of the image stack in three dimension.

## Discussion

### High-Resolution Analytical SEM for CLEM

Correlative LM combined with SEM and FIB-SEM is of fundamental importance to biomedical research. However, in order for this technique to evolve to a routinely used method, reproducible, efficient and cost-effective procedures need to be further developed. In this regard, compared with TEM, SEM has not reached the widespread use in bio-sciences that one might expect given its high resolution and potential. The potential of analytical high-resolution SEM techniques remains largely unexplored for many scientists starting out with FIB/SEM tomography. Published protocols may suggest that FIB/SEM milling is a stand-alone technique for correlative microscopy. However, we would argue that FIB/SEM milling is only one of the many facets of high-resolution SEM. Fully exhausting the benefits of all SEM capabilities is a prerequisite for efficient routine correlative microscopy. The right handling of WD, high voltage, the use and variation of different parameters (chamber SE, in-lens SE, in-lens EsB, QBSD, apertures, high-current mode) mean that SEM can be a versatile and powerful tool for a wide range of applications (Scala et al., 1985; Bozzola & Russell, 1991).

Correlation of light and EM as a method became substantially more elaborate with the introduction of serial block-face sectioning. Now, a 3D-LM data set with precise reference coordinates serves a basis (Lucas et al., 2012; Karreman et al., 2016; Cheng et al., 2017; Lees et al., 2017; Russell et al., 2017). For standard EM preparations, specimens have to be cut into small pieces, which is accompanied by a significant loss of the 3D-context. Even after oriented embedding, correlation can still be exquisitely difficult. The basic problem is that, in contrast to 2D, we are typically unable to correlate volumes without computing. Even our eyes do not see in 3D but rather interpret spaces by 2D images on the retina. The simple postulate is: correlate in 2D and make the specimen suitable for it.

Which geometry of biological samples is desirable or restrictive for CLEM? The main limitation is in the FIB/SEM operation. The maximum milling depth is about 100 *µ*m, but due to curtaining in at these depths, a more practical depth is around 50–60 *µ*m. Specimens within that range are suitable for LM. Thus, if cells or tissues are naturally within this range (bacteria: few *µ*m; HeLa cells: 20 *µ*m; diatoms/algal cells 5–100 *µ*m), then they simply have to be immobilized onto a slide. Depending on the sample, however, this can be quite challenging. If larger tissues are used (e.g. mouse brain with a volume of approx. 1 cm^3^), the classic way of cutting small cubes with a feed size of 1 mm may result in an astronomic number of pieces (e.g. ~1000, each with the risk of severe damage), which should be fixed and embedded numerically. Cutting the same volume with a vibratome into 50–70 *µ*m sections reduces the number of individual slices to 150–200. Compared with 1 mm^3^ cubes, these slices have, due to their minor thickness, several advantages: (i) they are suitable for all LM modes (bright field, DIC, fluorescence, CLSM); (ii) fixation and contrast enhancement is faster and more consistent due to more permissive diffusion conditions; (iii) the entire slice can be investigated with SEM at high resolution; (iv) each ROI defined in LM can be re-localized by simple triangulation, as LM and SEM images match perfectly (Fig. 9b).

### The Right Coordinate System

It is undisputed that a robust coordinate system is a solution for any correlative microscopy technique. This implies that instead of the structural details, the images of the coordinates in LM and SEM are initially correlated. This correlation is trivial, but only if the labels are clearly visible in both microscopy modalities (Fig. 5a). Today, complete solutions are commercially available and generally based on the same idea: selecting an ROI in LM, storing the coordinates, switching to SEM and recalling the coordinates (Liv et al., 2013; Brama et al., 2016; Schorb & Sieckmann, 2017). This is a valuable improvement if a set of instruments from the same manufacturer is used and trimming, mounting etc. does not alter the specimen. In practice, however, LM and SEM systems from different manufacturers, which do not intrinsically share a coordinate system, are often used for CLEM. To this end, inherent labels on the specimen are most beneficial for investigating the same specimen on different systems, for example when changing from LM instruments (LSM for fluorescence and DIC) to SEM or using micro-CT or X-ray microscopy. With different labeling methods covering a broad range of requirements for different applications, correlation with any microscope should be possible. Since the coordinate system is a 2D pattern, superimposition and only linear scaling or rotation is required—if at all. Finally, any image distortion is instantly recognized.

### Immobilization and Flat Embedding

A prerequisite for any flat embedding of cells, cell aggregates, and tissues is their immobilization in a fixed position relative to the coordinate system. Immobilization of different biological objects, capable of withstanding the entire EM procedure, including exchanging fixatives, solutions, washing steps, and dehydration in ethanol or acetone, will always be challenging. From a variety of available adhesives, we found two that particularly stand out. First, Biobond had very good adhesive properties for prokaryotic cells, larger eukaryotic cells and cell aggregates (Fig. 8). Since the adhesive coating is in the nm range, the evenness of the slide is maintained, which is important for high-resolution SEM, e.g. of cells with flagella or filopodia. Second, Cell-Tak^TM^ is the strongest adhesive and therefore the best choice for larger tissue sections. As Cell-Tak^TM^ is preferentially spread with a coverslip, the thickness cannot be precisely controlled, which limits its application in SEM for high-resolution topography of cell adherent structures (Fig. 9a). Although the chance for successful immobilization of any objects is on 50%, flat embedding is still possible by alternative strategies. If live-cell imaging is dispensable or is not possible (e.g. high-pressure freezing of non-adherent cells), samples can be processed until infiltration with resin, spread onto slides with coordinates and embedded ultra-thin or thin (Figs. 3d, 7d, 8b, 8c). CLEM is still possible as fluorescence can surprisingly still be detected for a variety of objects, although much weaker, after HPF, FS, and embedding (Fig. 8b). This signal provides practical information e.g. about vitality (vegetative cells of *Anabaena catenula*) or stages of cell differentiation (heterocysts of *A. catenula*) for a directed and efficient milling (Fig. 8). It is generally attractive to embed samples into resins which maintain fluorescence such as Lowicryl (Kukulski et al., 2012). However, it must be determined experimentally whether this rather soft resin is stable enough for high-resolution FIB/SEM. If samples are (i) available only in few individuals; (ii) fragile; (iii) or have to be kept flat during fixation and dehydration (histological sections), the presented filter system is of great benefit (Fig. 3f).

**Figure 12:**
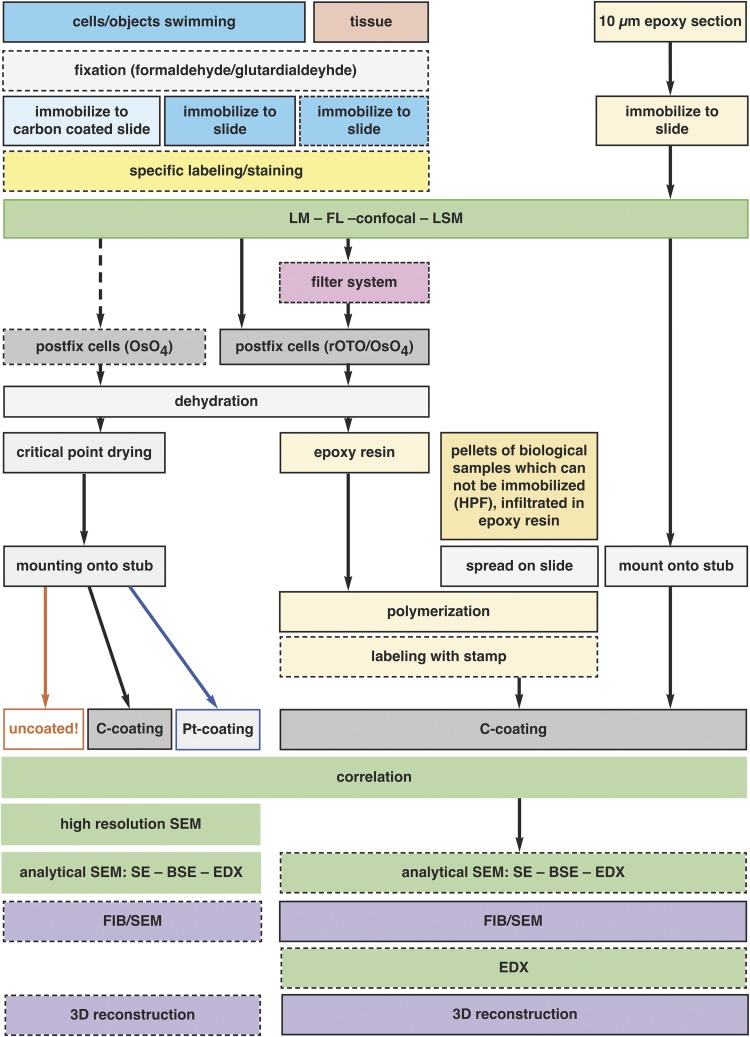
Preparation for correlative light and electron microscopy (CLEM). The flow chart illustrates the general schemes for the preparation of various specimens for flat embedding. The diagram includes all common specimen types from single cells to tissue sections, adherent cells, several immobilization procedures, and preparation of specimens which cannot be immobilized in principle (e.g. HPF frozen living cells), conservative fixation and embedding of fragile objects with a filter system and pre- and post-embedding CLEM. The possibilities of analytical investigations with the corresponding preparation or instrumental conditions are indicated.

CLEM is limited, but still possible for histological sections, as their characteristic shape and surface features are retained and can be examined after thin/ultra-thin embedding with LM and SEM (Fig. 9). The necessity and advantage of thin embedded samples became a topic of discussion within the last years, with the increasing demand of FIB/SEM in biosciences. A few protocols are published for flat embedding in resin by draining, blotting, centrifugation with the primary aim to reduce the resin layer to a minimum (Kizilyaprak et al., 2014; Lucas et al., 2017; Schieber et al., 2017). In practice, however, the aim is not an ultimately thin epoxy resin layer, but rather to control the thickness, depending on the samples and the scientific question. The main obstacle to this control is that the viscosity of resin rises significantly on the seconds to minutes timescale during spreading into thin layers, resulting in non-reproducible thickness. The removal of excessive resin by ethanol (Belu et al., 2016) is a good attempt but bears the risk of uncontrolled reduction, which is critical, when prokaryotic cells should be embedded in a resin layer of only a few microns thick. By keeping the slides in an acetone-saturated chamber (Fig. 3c), the fluidity of the resin is retained, until an even spreading is achieved. By changing the parameters (surface properties of slides or cover slips, resin concentration, draining by gravity, centrifugation, exposure time), a suitable thickness of resin can be achieved for individual samples after a few test runs. We illustrate the multiple possibilities for immobilization, fixation and thin embedding of various specimens for CLEM in a flow chart (Fig. 12).

### Thick Sections for Bridging TEM-Tomography with FIB/SEM

When comparing TEM- and FIB/SEM tomography, the excellent TEM resolution in *xy* cannot be reached with SEM due to physical and geometrical considerations (Giannuzzi, 2004). Section thickness for TEM-tomography, however, is limited to ~200–1000 nm, ideal for prokaryotic cells and cellular structures as the endoplasmic reticulum, but not for the complex 3D architecture of entire eukaryotic cells (Ercius et al., 2015). TEM-tomography followed by FIB/SEM tomography is an attractive solution to combine the highest resolution with very large volume information. This works reliable if a 200–400 nm section from a resin block is used for TEM-tomography and the following 10 *µ*m section is used for FIB/SEM of the same ROI, which can be repeated several times in cycles. Within a very large 3D volume (e.g. an entire cell), high-resolution 3D details can be implemented. The potential of thick serial sections for FIB/SEM is impressively demonstrated by Hayworth et al. (2015).

### Defining Space

Three orthogonal planes define a cube, typically achieved by FIB/SEM milling, resulting in an image stack. After milling, the defined planes are not maintained due to specimen drift, image distortions etc. (Schaffer et al., 2007; Boergens & Denk, 2013; Šedivý & Jäger, 2017; Storm et al., 2017). Therefore, an alignment of each image stack is necessary, which requires absolute fiducial markers for reference. The block face image represents the *xy* plane. If image stacks of resin blocks are automatically aligned (without references), the alignment is optimistically assumed to be correct, as it cannot be verified easily, except if substructures of known geometry (e.g. spheres) are present in abundance. The generation of references is not trivial for resin blocks. However, thin embedded samples provide the second reference plane in *xz* automatically, given by the surface of the slide/coverslip (Figs. 5f–5h; 11). The mandatory third reference plane can be easily produced, by milling parallel “lines”, which represent *de facto* vertical planes through the resin, ending orthogonally in the glass slide (Figs. 5h; 11j). In fact, most FIB/SEM image stacks require drift correction, and thus reliable references are necessary.

Independent of the method used for alignment of a FIB/SEM stack (Kreshuk et al., 2011; Cardona et al., 2012; Saalfeld et al., 2012; Schindelin et al., 2012), the accuracy can be validated by the reference coordinates and adjusted manually, which is likely necessary for long series (Han et al., 2018). Without references, any changes in volume geometry cannot be recognized or corrected (Storm et al., 2017). There are promising examples of intelligent software which aim to learn each step of alignment by mimicking the human approach (Sommer & Gerlich, 2013; Kraus & Frey, 2016; Kan, 2017). These programs, however, require substantial computing power and are still far away from being routinely applicable to large data sets.

### Cost-Effectiveness Considerations

If state of the art techniques is established as routine methods, cost-effectiveness becomes an important consideration. FIB/SEM will be always time-consuming, due to the enormous number of images for high-resolution tomography. Numerous scientific questions lead to results to some extent by chance, for instance when pellets of microorganisms are milled (Fig. 11g). In this regard, obtaining a longitudinal section through a heterocyst of cyanobacteria between vegetative cells, at the right stage and in the right orientation, is like winning the lottery. With flat embedded cyanobacteria filaments this can be achieved routinely with all controls, using different strains and culturing conditions (Fig. 8).

For questions involving eukaryotic cells or tissues, several efficiency aspects have to be considered. For example, for a HeLa cell in metaphase (spherical, with a diameter of 20 *µ*m), a cube with a 20 *µ*m feed size has to be milled. Then, by setting the section thicknesses to 10 nm, 2000 sections are needed. Under best conditions, with rOTO contrast enhancement (Seligman et al., 1966; Willingham & Rutherford, 1984) and using the in-lens SE-detector, the exposure time for an acceptable block face image can be around 15 s. This results in roughly an 8 h exposure time for the whole cell, thereby possible within a day. Classical glutaraldehyde/osmiumtetroxide (Palade, 1952) fixation, however, gives a much weaker contrast. If using the EsB detector, an exposure time of one minute or more is expected. Thus, the FIB/SEM experiment becomes a 2-day venture with at least one overnight session, bearing risks of the instability of the system, heating of the Ga-emitter and loss of information during the restart of the milling procedure. For a HeLa cell, milling time is much less than the exposure time. However, for cross sections of *C. elegans*, the milling time increases dramatically, especially when low ion currents (100–200 pA) have to be used. FIB/SEM investigations increase to several days or even weeks with costs of several thousand dollars per run.

The costs for FIB consumables (Ga-emitter, apertures, service costs) can be reduced to 50% simply by flat embedding due to omitting a ramp and reducing the volume to be milled exclusively to the ROI (compare: Fig. 11g with 11i). Beside economic aspects, an important benefit of flat embedded samples, compared with established procedures, is (i) saving time; (ii) the possibility of setting the milling frame as precisely as desired for cross or longitudinal section; and (iii) starting close to structural details documented before in LM, e.g. cellular inclusion, centrosomes etc.

## Summary

We developed an easy and broadly applicable procedure, consisting of several technical improvements of relevant scientific investigations, including the whole range of simple correlative, high-resolution LM/SEM microscopy, with immuno-labeling, flat embedding for FIB/SEM and post-embedding investigations of specimens (Fig. 12). Since every sample has its own handling requirements and limitations, several adjustments based on sample properties and scientific question are offered (Fig. 12). Defining and maintaining coordinates of a target structure is the most important aspect for re-localization in SEM. With sample preparation, coordinate labeling, and with the right conductive coating, the analytical potential of the SEM with all detectable signals (SE, BSE, EDX) is of great advantage for correlative investigations.
